# Changes in dietary patterns among Bangladeshi adult population during the COVID-19 pandemic: A web-based cross-sectional study

**DOI:** 10.1016/j.heliyon.2022.e10349

**Published:** 2022-08-18

**Authors:** Md. Akhtarul Islam, Mst. Tanmin Nahar, S. M. Farhad Ibn Anik, Sutapa Dey Barna, Md. Tanvir Hossain

**Affiliations:** a Statistics Discipline, Science Engineering & Technology School, Khulna University, Khulna 9208, Bangladesh; b Department of Business Administration, International Standard University, 69 Mohakhali C/A, Dhaka 1212, Bangladesh; c Sociology Discipline, Social Science School, Khulna University, Khulna 9208, Bangladesh

**Keywords:** COVID-19, Lockdown dietary patterns, Physical activities, Bangladesh

## Abstract

**Background:**

The home confinement induced by the COVID-19 pandemic affects individuals’ mental wellbeing and increases unhealthy behaviors, such as minimum to no physical activity, overeating, and substance use.

**Objective:**

This study aimed to assess the changes in dietary patterns among the Bangladeshi adult population during the COVID-19 pandemic and identify their determinants.

**Methods:**

This web-based cross-sectional survey was carried out from 10–17 December 2020 using an e-questionnaire based on Google Forms. A semi-structured e-questionnaire was forwarded to the participants – Bangladesh citizens aged above 18 years – through social media platforms and email in order to collect information about socio-demographic issues and multidimensional dietary patterns. From the initial 817 responses gathered through snowball sampling, 748 responses were retained. Bivariate and multivariate analyses were executed.

**Results:**

The findings suggest that 50% of the participants reported a reduction in weight and physical activities, while approximately 52% experienced increased sleep time. One in three participants (31.4%) experienced a decrease in food buying capacity. The findings further indicate that women were 1.65 times more likely to reduce food consumption than men. Meanwhile, employed people were about 34% less likely to increase food consumption than their unemployed counterparts. People who were getting more than 6 h of sleep per day were nearly 61% less likely to increase food consumption than people who slept for less than 6 h per day. People struggling to buy food items were 2.31 times more likely to reduce food intake than people with no such limitations.

**Conclusions:**

The study shows that COVID-19 has substantially affected Bangladeshi people’s common food consumption patterns. Being confined within the household, primarily due to countrywide lockdowns and ‘general holidays’, has affected both the dietary patterns and the financial wellbeing of people. Therefore, the concerned authorities should promote effective nutrition education and healthy dietary behaviors; meanwhile, financial support or incentives for people in need are also strongly advocated.

## 1 Introduction

The global outbreak of the novel coronavirus disease 2019 (COVID-19), caused by Severe Acute Respiratory Syndrome Coronavirus-2 (SARS-CoV-2), first spread from Wuhan in China [1]. The World Health Organization (WHO) labeled the COVID-19 disease a pandemic on 11 March 2020 [2]. As a precaution, to limit the human-to-human spread of COVID-19, countries across the world implemented a range of non-therapeutic interventions such as restricting travel for foreigners, shutting down public places such as educational institutions and shopping malls, and closing entire transit systems [3, 4, 5], in the absence of appropriate measures such as vaccines.

In Bangladesh, the first case of COVID-19 was identified on 8 March 2020; hence, the government imposed a countrywide lockdown, in the disguise of ‘general holidays’, from 26 March 2020, in order to minimize the spread of COVID-19 at the community level [6, 7, 8]. Before the lockdown was imposed, all educational institutions in Bangladesh suspended all on-campus academic and administrative activities from 17 March 2020, which was later extended until mid-June 2021 [9]. Although online-based education was introduced in mid-2020, it was reportedly plagued by many problems [10]. The strict non-therapeutic measures implemented by the government during the lockdown had a significant impact not only on people’s mental wellbeing [5, 11, 12, 13, 14, 15] but also on their individual dietary patterns and lifestyle, including access to and availability of food, patterns of food consumption, and form and degree of physical exercise [16, 17].

It is undeniable that nutritious food is essential for maintaining a healthy lifestyle and strengthening the immune system, particularly during health crises such as the COVID-19 pandemic [18, 19]. Individuals with a well-proportioned diet tend to be in good health, with the ability to reduce obesity and other health-related problems. Additionally, active immune systems will decrease the likelihood of being infected with harmful and infectious diseases such as COVID-19 [19,20]. Prolonged lifespan and wellbeing are also significantly related to a balanced diet and a healthy lifestyle [21]. In the absence of suitable antidotal medicines against COVID-19, nutritional dietary supplements containing vitamins and other essential elements could be the best way to strengthen immunity, especially in adults [22]. However, the pandemic-induced lockdown has had severe implications for health issues, including irregular or unhealthy diets, absence of physical exercise, and substance use; all of these could increase the risk of being infected with the disease and also trigger fear, anxiety, depression, and other mental disorders [23, 24, 25, 26]. Anxiety, depression, and other negative emotions associated with COVID-19 could also lead to overeating, mostly food full of carbohydrates; namely ‘food cravings’ or ‘emotional eating’ [27, 28, 29]. Moreover, the sense of monotony due to prolonged confinement within the home can also be associated with overeating in order to avoid dullness or boredom [30, 31].

Aside from excessive food intake, lower levels of physical activity and increased obesity among people were already global health concerns [32, 33] before the ongoing COVID-19 pandemic. The failure to reduce overeating and physical inactivity has led to chronic and non-communicable diseases such as obesity, diabetes, cancer, and cardiovascular disease [34]. However, during the COVID-19 pandemic, more emphasis was placed on home-based exercises to maintain people’s physical health [35], as mobility outside the home was strictly discouraged. The rapid spread of the COVID-19 outbreak brought about a substantial change in diet patterns and lifestyle [36, 37]. An uncontrolled increase in unhealthy food consumption and limited physical activities could significantly impact overall health conditions and increase the risk of various diseases, as has already been reported in other developing countries [38, 39, 40]. Moreover, multiple studies on epidemics such as Ebola, SARS, and H1N1 have shown that anxieties about health, stress, and weak psychosocial efficacy are common during health emergencies, and changes in eating behavior are also noticeable among people affected by such conditions [41, 42, 43, 44, 45].

Previous research has shown that undesirable natural disasters also disrupt people’s regular dietary patterns [46, 47]. Recent studies have indicated that individuals with health anxiety suffer from eating disorders during the COVID-19 [48, 49]. However, changes in dietary patterns during the COVID-19 pandemic have remained unexplored in Bangladesh. There is no doubt that maintaining a healthy dietary pattern and lifestyle is crucial for avoiding the onset risk factors for long-term non-communicable diseases such as diabetes, cardiovascular disease, and cancer following an emergency such as the COVID-19 pandemic. Likewise, a group of studies has found a negative relationship between cancer death rate and adherence to the Mediterranean diet [50]. They show that maintaining a healthy diet is necessary for modulating the processing rate of anxiety and inflammation, because food influences different gene expression levels in cytokines [51]. This study, therefore, has aimed to explore the changes in dietary patterns among the Bangladeshi adult population during the COVID-19 pandemic and to determine the influencing factors of such changes during involuntary home confinement. The study is expected to provide Bangladesh’s policymakers and development stakeholders with a snapshot of the changing dietary patterns among the Bangladeshi population during the COVID-19 lockdown, using cross-sectional data. This study would help policymakers rethink existing health, development, and emergency strategies and enact new effective policies and strategies in order to avoid pandemic-induced food intake and related health crises.

## 2 Methods and materials

### 2.1 Data source and sampling techniques

Data for this web-based cross-sectional study was collected using an e-questionnaire based on Google Forms. The link of the semi-structured e-questionnaire, consisting of both open and closed-ended items, was shared on student community pages through social media such as Facebook, Messenger, and WhatsApp, and forwarded using other means, including emails. Data were collected using the snowball sampling technique, where the participants were requested to forward the link of the e-questionnaire to other individuals in their social network [5, 52, 53]. The participants were invited to complete the survey voluntarily. It is important to note that they were selected based on some specifications: i.e., each participant (i) must be a Bangladeshi citizen; (ii) must have been living in Bangladesh during the COVID-19 pandemic; (iii) must be 18 years of age or above; (iv) must have a valid social media or email account; and (v) must be able to read the e-questionnaire written in Bangla. A total of 817 individuals filled out the e-questionnaire voluntarily; 748 responses were retained for this study, as some responses were from individuals below 18 years of age, while other responses were discarded because they were incomplete.

### 2.2 Ethical issues

The Khulna University Ethical Clearance Committee (KUECC-2021/02/10) formally approved this study. The participants responded voluntarily and anonymously to the web-based survey by filling out an informed consent form affixed at the beginning of the e-questionnaire. In the consent form, all the participants were provided with information about the research purpose and assured of the confidentiality of the information and the right to revoke participation without any prior justification.

### 2.3 Questionnaire

The semi-structured e-questionnaire consisted of 32 questions within three distinct but mutually inclusive modules: section one contained questions regarding socio-demographic information; section two extracted information regarding lifestyle during the COVID-19 pandemic; and the third and final section focused on questions about dietary habits and practices during the COVID-19 pandemic [54]. The e-questionnaire was made available between 10 and 17 December 2020 for the participants to fill out. However, a pre-test with 30 participants was carried out beforehand in order to assess the validity and reliability of information extracted by the e-questionnaire. Following the pre-test, some minor adjustments were made to the e-questionnaire, and it was then made open to all, excluding the participants in the pre-test.

### 2.4 Measures

The dependent variable in this study was total food consumption status (such as snacks, sugar-sweetened beverages, confectionery, fruit, vegetables, regular meals, and drinking water) during COVID-19 compared to the pre-pandemic period. The participants were asked to reflect on their consumption of overall food intake behavior, and the responses were categorized into ‘No changes,’ ‘Decreased’, and ‘Increased’. They were then later re-categorized into two subgroups: (1) less consumption pattern versus constant consumption; and (2) greater consumption pattern versus constant consumption.

### 2.5 Socio-demographic information

Data were collected regarding age, gender, marital status, place of residence, division, employment status, living arrangements, body mass index (BMI), and education of participants during the COVID-19 pandemic. The participants’ age data were categorized into three groups as follows: (1) ≤25 years; (2) 26–35 years; and (3) ≥36 years. Employment status was re-categorized into: (1) unemployed people, who did not engage in work during the pandemic; and (2) employed people, who were engaged in work during the pandemic. Living arrangements during the pandemic were also classified into three groups: (1) living alone; (2) living with family; and (3) others. Individuals also reported information on height and weight (last measured before the pandemic and during the pandemic) to compute body mass index (BMI) by applying the Quetelet equation (body weight in kilograms/height squared in meters). BMI was then defined according to the guidelines of the World Health Organization (WHO) [55]. There were four classifications: underweight (BMI = below 18.5 kg/square meters); normal weight (BMI = 18.5 kg/square meters to 25.0 kg/square meters); overweight (BMI = 25.0 kg/square meters to 30.0 kg/square meters); and obese (BMI = 30.0 kg/square meters and above) [56].

### 2.6 Dietary issues

In the e-questionnaire, some questions referred to nutritional behavior during the COVID-19 pandemic. These questions assessed the proportions of food consumption, with each proportion considered as food intake. For example, for vegetable intake, 1 portion = 100g, and for fish intake, 1 portion = 100–150g. Sugar-sweetened beverage intake and water intake were measured by a glass equaling 250ml. There were also some relevant questions regarding changes in food consumption during the pre-COVID-19 period. The participants were asked for information regarding changes in intake of food such as snacks, sugar-sweetened beverages, confectionery, fruit, vegetables, regular meals, and drinking water; the available response options were ‘I ate more’, ‘I ate less’, ‘I ate the same’, and ‘I did not eat that item before or during the pandemic’. The participants also provided information regarding the consumption of dietary supplements or medications containing nutrients (e.g., vitamins, minerals, fatty acids). They were also requested to specify the reasons for any problem with their food item procurement, if applicable, and the answers were categorized into four categories: ‘economic instability’; ‘safety and transport’; ‘lack of work’; and ‘inadequacy and higher price’.

### 2.7 Lifestyle issues

The participants were requested to provide information regarding their lifestyle both pre- and during the COVID-19 pandemic. They answered questions about physical activity, screen time, and smoking habits. There were two questions regarding physical activity: one regarding the amount of time they spent on physical activity per day both at home and outside (e.g., walking, cycling, running); and other about changes in their physical activity during the COVID-19 pandemic. The participants were also asked about changes in their screen time during the COVID-19 pandemic; the answers were categorized as ‘increased’, ‘decreased’, and ‘has not changed’. The participants then gave their reasons for spending time in front of screens, such as ‘work’, ‘entertainment’, ‘boredom’, and ‘learning’. A question about sleep time per day (categorized into ‘up to 6 h’ and ‘more than 6 h’) was asked regarding changes in sleep time (available responses were ‘decreased’, ‘has not changed’, ‘increased’, and ‘I do not know’) in order to compare the situation pre- and during the COVID-19 pandemic. The participants were also asked about changes in their smoking habits during the pandemic.

### 2.8 Statistical analysis

Data were analyzed in three consecutive stages: univariate analyses descriptive statistics, i.e., percentage, mean, and standard deviation, was used to present the essential characteristics of the participants; bivariate analysis (Pearson's Chi-square [χ^2^]) assessed the association between dietary patterns and socioeconomic factors as well as lifestyle; and finally, multivariate analysis (binary logistic regression [BLR]) determined the extent to which socioeconomic and lifestyle factors were influencing the dietary patterns of Bangladeshi adults. There were two dependent variables: ‘consumption increased’, comprising ‘constant’ and ‘increased’; and ‘consumption decreased’, consisting of ‘constant’ and ‘decreased’. The statistical analyses were performed by IBM SPSS Statistics, version 27.

## 3 Results

From Table 1, it is evident that 58.4% of the participants were male, and around 53% were roughly 25 years of age. Among the participants, more than half (60.7%) were unmarried and resided in urban areas (57.8%), while around 84% lived with their families. Two out of five participants (44.9%) were undergraduate students residing within the Khulna division (43.7%). Nearly 26% of the participants were unemployed during the pandemic. However, during COVID-19, about 70% of the participants were normal weight, while 8.4%, 18.7%, and 2.9% of the participants were underweight, overweight, and obese, respectively. Before the pandemic, around 73% of the participants had been within the normal weight range (BMI = 18.5–24.9 kg/m^2^). The findings also show that about 25% of the participants reduced their snack intake (including sugar-sweetened beverages and confectionery products) and 14.7% decreased fruit intake, whereas 38% increased water intake and about 41% increased vegetable intake. Nearly half (47.1%) of the participants increased their food consumption, while nearly 23% experienced a reduction in food intake. About 39% of the participants increased their regular meal intake, while 47.1% had difficulties with food purchases. About 43% of the participants took dietary supplements or medications containing nutrients during the COVID-19 pandemic. Nearly 52% experienced an increase in sleeping time, and 81.8% slept more than 6 h per day. Meanwhile, more than half (67%) experienced an increase in screen time; 37.8% reported that entertainment was a reason for their increased screen time. Around 50% of the participants decreased their physical activities; 19.5% reduced smoking.

**Table 1:** Basic information.

Variables	Frequency	Percentages	Variables	Frequency	Percentages
**Socio-demographic information**					
**Gender**			**Age**		
Male	439	58.7	≤25 years	394	52.7
Female	309	41.3	26–35 years	167	22.3
**Marital status**			≥36 years	187	25.
Unmarried	454	60.7	**Living arrangement**		
Married	270	36.1	Living with family	624	83.4
Divorced	10	1.3	Living alone	113	15.1
Widowed	14	1.9	Others	11	1.5
**Education**			**Employment status during pandemic**		
Primary	31	4.1	Unemployed	190	25.4
Secondary	89	11.9	Employed	558	74.6
Higher secondary	197	26.3	**Place of residence**		
Undergraduate	336	44.9	Rural	316	42.2
Postgraduate	95	12.7	Urban	432	57.8
**BMI before COVID-19**			**Division**		
Below 18.5	74	10.0	Khulna	327	43.7
18.5–24.9	538	72.6	Dhaka	162	21.7
25.0–29.9	109	14.7	Chattogram	36	4.8
30 and above	20	2.7	Rajshahi	91	12.2
**BMI during COVID-19**			Barishal	35	4.7
Below 18.5	61	8.4	Rangpur	32	4.3
18.5–24.9	508	70	Sylhet	34	4.5
25.0–29.9	136	18.7	Mymensingh	31	4.1
30 and above	21	2.9			
***Dietary issues***					
**Changes in intake of snack, sugar-sweetened beverages, confectionery products**			**Changes in fruits intake**		
No changes	352	47.1	No changes	323	43.2
Decreased	140	18.7	Decreased	110	14.7
Increased	190	25.4	Increased	301	40.2
Do not eat at all	66	8.8	Do not eat at all	14	1.9
**Changes in drinking water**			**Changes in vegetable intake**		
No changes	395	52.8	No changes	350	46.8
Decreased	69	9.2	Decreased	78	10.4
Increased	284	38.0	Increased	303	40.5
**Changes in total food consumption**			Do not eat at all	17	2.3
No changes	226	30.2	**Regular meals intake changes**		
Decreased	170	22.7	No changes	297	39.7
Increased	362	47.1	Decreased	159	21.3
			Increased	292	39.0
**Having difficulties with food purchases**			**Intake of dietary supplements**		
No	513	68.6	No	430	57.5
Yes	235	31.4	Yes	318	42.5
***Lifestyle issues***					
**Sleep changes**			**Sleep per day**		
No changes	252	33.7	Up to 6 h	136	18.2
Decreased	85	11.4	More than 6hrs	612	81.8
Increased	388	51.9	**Reasons for increasing screen time**		
Do not know	23	3.1	Entertainment	283	37.8
**Change in screen time**			Work	167	22.3
No changes	165	22.1	Learning	146	19.5
Decreased	82	11.0	Boredom	152	20.3
Increased	501	67.0	**Smoking habits**		
**Changes in physical activity (PA)**			Non-smoker	494	66.0
Lower before and no changes	35	4.7	Decreased	146	19.5
Moderate before and no changes	198	26.5	Increased	69	9.2
Decreased	369	49.3	Others	39	5.2
Increased	146	19.5			

### 3.1 Association of dietary patterns with other covariates

Table 2 shows the Pearson’s Chi-square (χ^2^) test results, assessing the association between changing dietary patterns and socioeconomic and lifestyle factors. The findings suggest that 15 various covariates with initial statistical significance, namely age (p < 0.001), marital status (p = 0.011), employment status (p = 0.022), division (p = 0.049), changes in intake of snacks, sugar-sweetened beverages, and confectionery products (p < 0.001), changes in fruit intake (p < 0.001), changes in vegetable intake (p < 0.001), changes in regular meals (p < 0.001), changes in drinking water (p < 0.001), difficulties with food purchases (p = 0.002), changes in physical activity (PA) (p = 0.037), sleep changes (p = 0.010), smoking habits (p = 0.047), change in screen time (p = 0.006), and sleep per day (p = 0.007) were associated with a smaller consumption pattern as opposed to constant consumption. Again, the findings also suggest that 12 various covariates, namely BMI during COVID-19 (p = 0.002), living arrangements (p = 0.015), division (p = 0.044), changes in intake of snacks, sugar-sweetened beverages, and confectionery products (p < 0.001), changes in fruit intake (p < 0.001), changes in vegetable intake (p < 0.001), changes in regular meals (p < 0.001), changes in drinking water (p < 0.001), difficulties with food purchases (p < 0.001), changes in physical activity (PA) (p < 0.001), sleep changes (p < 0.001), change in screen time (p < 0.001), and sleep per day (p < 0.020), were significantly associated with a greater consumption pattern as opposed to constant consumption.

**Table 2:** Chi-square table showing the association of food consumption with various covariates.

Characteristics	Consumption Status		*p* value	Consumption Status		*p* value
	Constant n (%)	Less n (%)		Constant n (%)	More n (%)	
***Socio-demographic information***						
**Gender**						
Male	136 (34.4)	92 (23.2)	0.227	136 (23.5)	211 (36.5)	0.955
Female	90 (22.7)	78 (19.7)		90 (15.6)	141 (24.4)	
**Age**						
≤25 years	134 (33.8)	67 (16.9)	0.000	134 (23.2)	193 (33.4)	0.569
26–35 years	45 (11.4)	43 (10.8)		45 (7.8)	79 (13.7)	
≥36 years	47 (11.9)	60 (15.2)		47 (8.1)	80 (13.8)	
**BMI before COVID-19**						
Below 18.5	24 (6.1)	14 (3.6)	0.571	24 (4.2)	36 (6.3)	0.168
18.5–24.9	155 (39.7)	115 (29.4)		155 (27.1)	268 (46.9)	
25.0–29.9	34 (8.7)	34 (8.7)		34 (6.0)	41 (7.2)	
30 and above	8 (2.0)	7 (1.8)		8 (1.4)	5 (0.9)	
**BMI during COVID-19**						
Below 18.5	27 (7.1)	19 (5.0)	0.097	27 (4.8)	15 (2.7)	0.002
18.5–24.9	141 (37.1)	118 (31.0)		141 (25.0)	249 (44.2)	
25.0–29.9	39 (10.3)	25 (6.6)		39 (6.9)	72 (12.8)	
30 and above	10 (2.6)	1 (0.3)		10 (1.8)	10 (1.8)	
**Education**						
Primary	11 (2.8)	15 (3.8)	0.374	11 (1.9)	5 (0.9)	0.156
Secondary	26 (6.6)	21 (5.3)		26 (4.5)	42 (7.3)	
Higher secondary	60 (15.2)	49 (12.4)		60 (10.4)	88 (15.2)	
Undergraduate	101 (25.4)	62 (15.6)		101 (17.5)	173 (29.9)	
Postgraduate	28 (7.1)	23 (5.8)		28 (4.8)	44 (7.6)	
**Marital status**						
Unmarried and others	153 (38.7)	90 (22.7)	0.011	153 (26.6)	235 (40.6)	0.957
Married	73 (18.4)	80 (20.2)		73 (12.6)	117 (20.2)	
**Living arrangement**						
Living with family	183 (46.2)	141 (35.5)	0.686	183 (31.8)	300 (51.9)	0.015
Living alone	36 (9.1)	26 (6.6)		36 (6.3)	51 (8.8)	
Others	7 (1.8)	3 (0.8)		7 (1)	1 (0.2)	
**Employment status during pandemic**						
Unemployed	51 (12.9)	41 (10.4)	0.022	51 (8.8)	98 (17.0)	0.412
Employed	175 (44.1)	129 (32.6)		175 (30.2)	254 (44)	
**Place of residence**						
Rural	90 (22.7)	77 (19.4)	0.275	90 (15.6)	149 (25.8)	0.550
Urban	136 (34.4)	93 (23.5)		136 (23.5)	203 (35.1)	
**Division**						
Khulna	95 (24.1)	76 (19.2)	0.049	95 (16.4)	156 (27.0)	0.044
Dhaka	69 (17.4)	27 (6.8)		69 (11.9)	66 (11.4)	
Chattogram	10 (2.5)	7 (1.8)		10 (1.8)	19 (3.3)	
Rajshahi	23 (5.8)	25 (6.3)		23 (4.0)	43 (7.4)	
Barishal	9 (2.3)	10 (2.5)		9 (1.6)	16 (2.8)	
Rangpur	6 (1.5)	7 (1.8)		6 (1.0)	19 (3.3)	
Sylhet	6 (1.5)	8 (2.0)		6 (1.0)	20 (3.5)	
Mymensingh	8 (2.0)	10 (2.5)		8 (1.4)	13 (2.2)	
***Dietary issues***						
**Changes in intake of snack, sugar-sweetened beverages, confectionery products**						
No changes	161 (40.7)	71 (17.9)	0.000	161 (27.9)	120 (20.9)	0.000
Decreased	28 (7.1)	59 (14.9)		28 (4.8)	53 (9.2)	
Increased	16 (4.0)	18 (4.5)		16 (2.8)	156 (27.0)	
Do not eat at all	21 (5.3)	22 (5.6)		21 (3.6)	23 (4.0)	
**Changes in fruits intake**						
No changes	152 (38.4)	62 (15.6)	0.000	152 (26.3)	109 (18.9)	0.000
Decreased	17 (4.3)	64 (16.1)		17 (2.9)	29 (5.0)	
Increased	52 (13.1)	43 (10.9)		52 (9.0)	206 (35.6)	
Do not eat at all	5 (1.3)	1 (0.3)		5 (0.9)	8 (1.4)	
**Changes in vegetable intake**						
No changes	160 (40.4)	63 (15.9)	0.000	160 (27.7)	127 (22.0)	0.000
Decreased	10 (2.5)	38 (9.6)		10 (1.7)	30 (5.2)	
Increased	53 (13.4)	65 (16.4)		53 (9.2)	185 (32.0)	
Do not eat at all	3 (0.8)	4 (1.0)		3 (0.5)	10 (1.7)	
**Changes in regular meals**						
No changes	170 (42.9)	31 (7.8)	0.000	170 (29.4)	96 (16.6)	0.000
Decreased	19 (4.9)	116 (29.3)		19 (3.3)	24 (4.2)	
Increased	37 (9.3)	23 (5.8)		37 (6.4)	232 (40.1)	
**Changes in drinking water**						
No changes	158 (39.9)	85 (21.5)	0.000	158 (27.3)	152 (26.3)	0.000
Decreased	11 (2.8)	33 (8.3)		11 (1.9)	25 (4.3)	
Increased	57 (14.4)	52 (13.1)		57 (9.9)	175 (30.3)	
**Intake of dietary supplements**						
No	137 (34.6)	96 (24.2)	0.406	137 (23.7)	197 (34.1)	0.269
Yes	89 (22.5)	74 (18.7)		89 (15.4)	155 (26.8)	
**Having difficulties with foods purchases**						
No	157 (39.6%)	92 (23.2%)	0.002	157 (27.2%)	264 (45.7%)	0.014
Yes	69 (17.4%)	78 (19.7%)		69 (11.9%)	88 (15.2%)	
***Lifestyle issues***						
**Changes in physical activity (PA)**						
Lower before and no changes	13 (3.3)	10 (2.5)	0.037	13 (2.2)	12 (2.1)	0.000
Moderate before and no changes	77 (19.4)	36 (9.1)		77 (13.3)	85 (14.7)	
Decreased	86 (21.7)	83 (21.0)		86 (14.9)	200 (34.6)	
Increased	50 (12.6)	41 (10.4)		50 (8.7)	55 (9.5)	
**Sleep changes**						
No changes	107 (27.0)	64 (16.2)	0.010	107 (18.6)	81 (14.0)	0.000
Decreased	25 (6.3)	31 (7.8)		25 (4.3)	29 (5.0)	
Increased	80 (20.2)	72 (18.2)		80 (13.8)	236 (40.8)	
Do not know	14 (3.5)	3 (0.8)		14 (2.4)	6 (1.1)	
**Smoking habits**						
Non-smoker	166 (41.9)	103 (26.0)	0.047	166 (28.7)	225 (38.9)	0.059
Decreased	31 (7.8)	36 (9.1)		31 (5.4)	79 (13.7)	
Increased	18 (4.5)	22 (5.6)		18 (3.1)	29 (5.0)	
Others	11 (2.8)	9 (2.3)		11 (1.9)	19 (3.3)	
**Change in screen time**						
No changes	75 (18.9)	39 (9.8)	0.006	75 (13.0)	51 (8.8)	0.000
Decreased	18 (4.56)	29 (7.3)		18 (3.1)	35 (6.1)	
Increased	133 (33.6)	102 (25.8)		133 (23.0)	266 (46.0)	
**Reasons for increasing screen time**						
Entertainment	93 (23.5)	60 (15.2)	0.159	93 (16.1)	130 (22.5)	0.735
Work	51 (12.9)	30 (7.5)		51 (8.8)	86 (14.9)	
Learning	40 (10.1)	35 (8.8)		40 (6.9)	71 (12.3)	
Boredom	42 (10.6)	45 (11.4)		42 (7.3)	65 (11.2)	
**Sleep per day**						
Up to 6 h	34 (8.6)	49 (12.4)	0.007	34 (5.9)	53 (9.2)	0.020
More than 6 h	192 (48.5)	121 (30.5)		192 (33.3)	299 (51.6)	

### 3.2 Predictors of changing dietary patterns

We have utilized the Binary Logistic Regression (BLR) model to show the adjusted odds ratios (with 95% confidence intervals) that have been adjusted to account for additional independent variables in the model. This model specified the determinants of changing dietary patterns among adults in Bangladesh during the COVID-19 pandemic. The findings, presented in Table 3, indicate that women were 1.65 times more likely to decrease their food consumption (AOR = 1.647, CI: 1.041–2.606) compared to men. The odds ratio corresponding to people aged ≥36 years of age was 96% (AOR = 1.962, CI: 1.044–3.686), suggesting that they were more likely to reduce their food intake than people aged ≤25 years of age. Employed participants were less likely to increase their total food intake (AOR = 0.654, CI: 0.427–1.001) than unemployed participants. People who had difficulties purchasing food during the pandemic had a 2.31 (AOR = 2.316, CI: 1.458–3.678) times higher probability of reduced food intake compared to those with no difficulties in purchasing food; meanwhile, they had a lower probability of increasing food intake (AOR = 0.705. CI: 0.457–1.047) compared to people with an abundance of food during the pandemic. People taking dietary supplements or medications containing nutrients had a 1.168 (AOR = 1.168, CI: 0.806–1.695) times higher probability of decreasing food intake compared to those who did not take dietary supplements or medications containing nutrients. Similarly, the participants with undergraduate education had a 3.27 (AOR = 3.266, CI: 1.459–7.310) times higher chance of increased food intake compared to those with no education. Furthermore, the participants who slept more than 6 h per day were less likely to reduce their food consumption (AOR = 0.393, CI: 0.233–0.666) than participants who slept equal to or more than 6 h per day.

**Table 3:** Results of Binary logistic regression model predicting total food consumption increased and decreased.

Variables	Total Consumptions Reduce				Total Consumptions Increased			
	AOR	*p* value	95% CI		AOR	*p* value	95% CI	
			Lower	Upper			Lower	Upper
**Gender**								
Male^r^								
Female	1.647	0.033	1.041	2.606	0.929	0.069	0.646	1.337
**Age**								
≤25^r^								
26–35 years	1.693	0.105	0.895	3.201	1.075	0.079	0.630	1.832
≥36	1.962	0.036	1.044	3.686	1.116	0.697	0.642	1.940
**Marital status**								
Married^r^								
Others	0.903	0.712	0.525	1.554	1.076	0.760	0.672	1.723
**Employment status during pandemic**								
Unemployed^r^								
Employed	0.971	0.915	0.057	1.655	0.654	0.050	0.427	1.001
**Living arrangement**								
Live Alone^r^								
Live with family or others	1.087	0.778	0.608	1.944	0.928	0.768	0.566	1.521
**Body mass index**								
Normal weight^r^								
Over or underweight	1.101	0.684	0.069	1.751	0.706	0.081	0.477	1.044
**Place of residence**								
Rural^r^								
Urban	1.049	0.849	0.640	1.721	0.956	0.821	0.645	1.416
**Division**								
Khulna^r^		0.209						
Dhaka	0.464	0.013	0.254	0.848	0.640	0.060	0.402	1.019
Chattogram	0.491	0.206	0.163	1.480	1.278	0.570	0.548	2.978
Rajshahi	1.051	0.886	0.531	2.082	1.123	0.699	0.624	2.022
Barishal	1.276	0.635	0.466	3.495	0.967	0.940	0.400	2.338
Rangpur	1.458	0.547	0.428	4.972	1.771	0.253	0.665	4.714
Sylhet	0.919	0.891	0.276	3.065	2.125	0.151	0.761	5.936
Mymensingh	1.238	0.694	0.427	3.586	1.037	0.942	0.391	2.747
**Having difficulties with foods purchases**								
No^r^								
Yes	2.316	0.000	1.458	3.678	0.705	0.083	0.475	1.047
**Taking dietary supplements or medications containing nutrients**								
No^r^								
Yes	0.920	0.724	0.057	1.463	1.168	0.041	0.806	1.695
**Education**								
Primary^r^		0.803						
Secondary	0.677	0.416	0.264	1.734	2.851	0.023	1.152	7.053
Higher secondary	0.937	0.885	0.388	2.260	2.751	0.017	1.194	6.338
Undergraduate	0.755	0.518	0.322	1.771	3.266	0.004	1.459	7.310
Postgraduate	0.680	0.431	0.260	1.779	2.867	0.028	1.122	7.326
**Sleep per day**								
UP to 6 h^r^								
More than 6 h	0.393	0.001	0.233	0.666	0.882	0.610	0.541	1.439

Notes: AOR. Adjusted odds ratio; CI. Confidence interval; r. Reference group.

## 4 Discussion

The COVID-19 pandemic has become a significant public health concern and has altered people’s daily routines worldwide. This pandemic has caused substantial changes in peoples’ lives, triggering an unparalleled effect on social life, lifestyle, health, and dietary patterns [57, 58]. This study has revealed that the COVID-19 pandemic has negatively affected the Bangladeshi adult population’s dietary patterns and relevant lifestyle. The study has found that changes in food intake were related to gender, age, employment status, education, BMI, intake of dietary supplements or medications containing nutrients, financial capacity to buy necessary food, level of education, and sleep time per day.

This study found that nearly half of female participants had higher chances of changing dietary patterns than their male counterparts. Research shows similar results in the United Arab Emirates, with women being more likely to change their dietary patterns than men [59]. Another study in Kuwait found that sex differences in psychological states and problems induced by the pandemic can be attributable to greater changes in dietary patterns among women compared to men [60]. Generally, the eating habits of nutrient intake are associated with familial interactions around food and weight issues, especially among women, considering their socio-cultural barriers, self-efficacy, and subjective norms compared to men [61, 62, 63]. Women are more likely to be involved in food preparation and other food-related activities. Consequently, this plays a crucial role in the food consumption behavior of women compared to men [62, 64]. Although this study found a significant change in dietary patterns between the sexes, there was no significant association between residence and dietary patterns during the COVID-19 confinement. A survey on Mediterranean regions, in contrast, found substantial differences in dietary behavior between urbanites and ruralites before and during the COVID-19 pandemic [65].

The findings of this study show that people over 36 years of age reduced their food intake to a greater degree than their younger counterparts (≤25 years). This change in dietary patterns could have been prompted by anxiety and fear among younger people, primarily from their exposure to social media and other misinformation sources [18, 66, 67, 68]. Studies suggest that many people under tremendous stress, depression, and emotional turbulence avoid food, or in some cases, increase their amount of food consumption [34, 69]. Another study, in contrast, found a negative association between age and dietary patterns [70]. For older people, a reduced food intake could be caused by the higher risk of complications and severity of COVID-19 among the older population [71, 72]. However, an extensive study is recommended to uncover the dynamics of age and food intake during health and similar other emergencies in the future, particularly in Bangladesh. Moreover, different nutrition promotion and awareness activities are also needed for the older population from lower socioeconomic backgrounds, in order to promote healthy dietary patterns among this population [70]. It would improve people’s immune systems and reduce their anxiety, stress and fear regarding COVID-19.

From this study, it is also evident that educated people have eaten more food than the non-educated during the pandemic. This finding is complemented by another study suggesting that increased dietary patterns are strongly associated with educational status [70]. Educated people generally have more secured job with higher and stable incomes and thus greater buying capacity. They also have more knowledge about nutrition, for instance in choosing different foods, understanding food labels, and choosing alternative foods from various food items to strengthen their immune system against diseases; these behaviours may be less common among people with low or no education [73].

This study also shows that participants with longer sleep time experienced a reduction in food intake during the pandemic. This result echoes a study that found that longer sleep time changed daily eating habits among the Croats during the pandemic [74]. In contrast, another study indicated that a reduction in sleep time significantly increases dietary change patterns [75]. To ensure a better lifestyle and dietary behavior during the pandemic, it is essential to provide information regarding COVID-19 and healthy lifestyle via the internet and health associates to the masses and to communities [76, 77].

Aside from sleep issues, this cross-sectional survey also found that people who had difficulties buying food items during the pandemic had to reduce food consumption. It has been well documented that the monetary crisis, in the forms of unemployment or underemployment and problems with food and price hikes, may have contributed to a reduction in buying capacity during the ongoing pandemic [15, 78, 79]. Furthermore, food purchasing behaviors depend on different sources of information, such as illness and healthcare, food market conditions and supply, and perceived mental health and confidence [78, 80, 81]. It has been more crucial during the pandemic to maintain an uninterrupted food supply and reduce food wastage in order to deal with these unprecedented events [82].

Furthermore, it was forecast that the COVID-19 pandemic might disrupt the availability and accessibility of food items because of the restrictions imposed on the transportation system, leading to higher food insecurity [83]. The findings from this study suggest that unemployed individuals experienced more significant changes in dietary patterns than the employed. Globally, small and medium enterprises worldwide have experienced a reduction of income and thereby been forced to dismiss their employees [78, 79], triggering a vicious cycle of financial crisis [15, 84].

Previous studies have shown a significant association between living arrangements/spatial locations and dietary patterns during the pandemic [54, 85]. This study, however, did not find any statistical significant association between living arrangements/spatial locations and dietary patterns.

### 4.1 Strengths and limitations

In this study, we assessed the changing patterns of dietary behavior of the adult Bangladeshi population using a web-based survey. This unique approach was popularized and proved effective during the COVID-19 pandemic as it allowed a prompt assessment of social phenomena. This study, carried out by administering an e-questionnaire, also allowed the collection of an impressive amount of information regarding people’s dietary patterns and healthy lifestyle behaviors before and during the lockdown, helping to predict changes in dietary patterns during the ongoing pandemic. The e-questionnaire was administered in Bangla – the native language of the Bangladeshi people – to ensure comprehensive understanding and spontaneous responses for each question; therefore, reliable information from the participants was assured. However, it also should be acknowledged that this study has several limitations. Although many responses were recorded, the variables were not elaborately explained, limiting the potential to compare each variable’s pre- and during-confinement effects. Furthermore, the sample might not represent the whole population of Bangladesh due to the application of the non-probability sampling approach. The study also relied on a web-based survey, where all data was self-reported by participants with access to the internet, thus excluding individuals with no or low access to the internet. Causality cannot be inferred under this investigation, since the characteristics of the comparable cross-sectional study design were considered using a snowball non-discriminatory sampling procedure. Due to sampling biases, the number of young adults was higher among the participants. Moreover, this study relied on the self-assessment of participants regarding changes in their diet and quality of life both pre- and during COVID-19; therefore, the study findings may not be generalizable due to recall bias. Hence, we recommend more extensive studies based on nationally representative samples and face-to-face interviews in order to understand the dynamics of changing dietary patterns during the COVID-19 pandemic.

## 5 Conclusion

The COVID-19 pandemic has made substantial changes to the life and living of people across the globe. The findings of this study indicate a negative impact of the pandemic on dietary patterns and other relevant issues, including physical activities, sleep, and screen time. It has been well documented that some people have struggled to afford necessary amenities, including food for their families, because of their financial constraints and limited knowledge of the nutritional properties of food items. Therefore, it is strongly recommended that countrywide awareness programs be initiated, using social and mass media, in order to make people aware of healthy dietary patterns and lifestyles. Moreover, along with its development stakeholders, the government should plan and implement policies and strategies to reach out to marginalized people in need of financial support, both in cities and other remote areas, particularly those involved in informal economic activities. Policymakers should also devise an inclusive and holistic approach to support and help the marginal social classes to recover from this unprecedented health emergency; this could be the only gateway for progress and development in the twenty-first century. Furthermore, more empirical and extensive research should be carried out in order to explore the changing patterns of dietary behavior among people, particularly in marginal communities in Bangladesh and other countries.

## Declarations

### Author contribution statement

Akhtarul Islam, Tanmin Nahar: Conceived and designed the experiments; Performed the experiments; Analyzed and interpreted the data; Contributed reagents, materials, analysis tools or data; Wrote the paper.

S. M. Farhad Ibn Anik: Conceived and designed the experiments; Contributed reagents, materials, analysis tools or data; Wrote the paper.

Sutapa Dey Barna: Contributed reagents, materials, analysis tools or data; Wrote the paper.

Md. Tanvir Hossain: Conceived and designed the experiments; Contributed reagents, materials, analysis tools or data; Wrote the paper.

### Funding statement

This research did not receive any specific grant from funding agencies in the public, commercial, or not-for-profit sectors.

### Data availability statement

Data associated with this study has been deposited at Harvard Dataverse under the accession number 10.7910/DVN/1SICDE.

### Declaration of interest’s statement

The authors declare no conflict of interest.

### Additional information

No additional information is available for this paper.
