# Corrigendum to “Changes in dietary patterns among Bangladeshi adult population during the COVID-19 pandemic: A web-based cross-sectional study” [Heliyon Volume 8, Issue 8, August 2023, Article e10349]

**DOI:** 10.1016/j.heliyon.2025.e43372

**Published:** 2025-05-07

**Authors:** Md Akhtarul Islam, Mst Tanmin Nahar, S.M.Farhad Ibn Anik, Sutapa Dey Barna, Md Tanvir Hossain

**Affiliations:** a Statistics Discipline, Science Engineering & Technology School, Khulna University, Khulna, 9208, Bangladesh; b Department of Business Administration, International Standard University, 69 Mohakhali C/A, Dhaka, 1212, Bangladesh; c Sociology Discipline, Social Science School, Khulna University, Khulna, 9208, Bangladesh

In the original published version of this article, there were irrelevant citation statement to the references.

The original manuscript showed the citation as:

Globally, small and medium enterprises worldwide have experienced a reduction of income and thereby been forced to dismiss their employees [78, 79]

The citation statement has been updated in the revised version of the article.

The corrected version of the citation can be found below:

Likewise, small and medium enterprises depending on forest resources in Bangladesh have experienced a reduction of income and thereby been forced to dismiss their employees [78, 79]

The authors apologize for the errors.

## Declaration of competing interest

The authors declare no conflict of interest.

